# Dr. Jenner, in Reply to Mr. Fermor

**Published:** 1801-10

**Authors:** Edw. Jenner

**Affiliations:** Cheltenham


### Dr. Jenner, *in Reply to* Mr. Fermor.

Dear Sir,

Accept my beſt thanks for your obliging letter, encloſing one from Dr. Wall on the ſubject of Vaccine Inoculation, the practice of which, it ſeems, has received a check at Oxford by ſome recent events there.

I ſhould certainly flatter myſelf too much, were I to conceive that a perfect knowledge of it could keep pace with the rapid progreſs it is making through the world. Miſtakes have happened both at home and abroad, and unqueſtionably will happen. The Vaccine lancet is not to be trifled with; and experience has taught us, that the Variolous ſhould be uſed with equal circumſpection. Where is the village that hath not yielded its victim to the Small-pox after inoculation for that diſeaſe was ſuppoſed to have been properly performed? Can the metropolis, even among the higher and more experienced Gentlemen of the medical Profeſſion, claim an exemption from fatal errors? Certainly not. Are we then, my dear Sir, when we conſider that it is impoſſible perfectly to underſtand the nature of the Cow-pox from a ſhort or ſuperficial examination, to be ſurprized at what has happened at Oxford, and ſome other places in our iſland? Dr. Wall was kind enough to conduct me to Green's, and there I luckily recollected a circumſtance that afforded me much ſatisfaction. The child that took the Small-pox after the Vaccine Inoculation, was the identical one that I chanced to ſee ſoon after it had been inoculated in the ſpring of laſt year. I then poſitively pronounced it inſecure, from the appearance of the arm, which by no means preſented the characteriſtic teſt. This caſe then, on which I found much ſtreſs had been laid, inſtead of involving the ſubject in doubt and obſcurity, throws upon it a blaze of light, for the reſt of Green's children (no inconſiderable number) who had gone through the Cow-pox perfectly, were expoſed to the infectious vapours of the Small-pox during its whole progreſs upon the child that caught it. The other patients, Slater's children, I did not ſee; had I ſeen them, it is probable ſome material deviation from the ordinary ſtate of the puſtule on the arm might have been perceptible. But, for the ſake of argument, let us ſuppoſe that one caſe in a thouſand, although the appearances on the arm be correct, ſhall, nevertheleſs, turn out unfavorable to our doctrine, the chance that two ſhall prove ſo *in ſucceſſion*, inoculated by the ſame perſon and with the ſame virus, is almoſt beyond calculation.

Had

Had I not met with inceſſant interruptions, I ſhould before this time have publiſhed a fourth paper upon the ſubject of the Cow-pox, deſcribing minutely that mode which experience has pointed out to me as the beſt for conducting its inoculation. But however diffuſe I may be in that Paper, the moſt material part may be compreſſed in the following conciſe ſentences. *Let the Vaccine fluid be taken, for the purpoſe of inoculation, from a perfect puſtule on ſome early day of its formation; I prefer the 5th, 6th, 7th, 8th, and (if the efflorescence is not far advanced beyond the margin of the puſtule) the 9th.* Making this my general principle, I never find any eſſential deviation in the character of the Vaccine puſtule in conſtitutions fully ſuſceptible of the action of the virus.

I feel extremely obliged by the unremitting attention you give to this important ſubject, and remain, with much eſteem,

Dear Sir,

Your faithful humble ſervant,

*Cheltenham,* 7 *Sept.* 1801. EDW. JENNER.

